# FAM83A is a potential biomarker for breast cancer initiation

**DOI:** 10.1186/s40364-022-00353-9

**Published:** 2022-02-19

**Authors:** Natascia Marino, Rana German, Ram Podicheti, Pam Rockey, George E. Sandusky, Constance J. Temm, Harikrishna Nakshatri, Rebekah J. Addison, Bryce Selman, Sandra K. Althouse, Anna Maria V. Storniolo

**Affiliations:** 1 Susan G. Komen Tissue Bank at the IU Simon Comprehensive Cancer Center, Indianapolis, IN 46202 USA; 2 Department of Medicine, Indiana University School of Medicine, Indianapolis, IN 46202 USA; 3 Center for Genomics and Bioinformatics, Indiana University, Bloomington, IN 47405 USA; 4 Pathology and Laboratory Medicine, Indiana University School of Medicine, Indianapolis, IN 46202 USA; 5 Department of Surgery, Indiana University School of Medicine, Indianapolis, IN 46202 USA; 6 Department of Biostatistics and Health Data Sciences, Indiana University School of Medicine, Indianapolis, IN 46202 USA

**Keywords:** Normal breast, FAM83A, Breast cancer, Cell transformation

## Abstract

**Background:**

Family with sequence similarity 83 member A (FAM83A) presents oncogenic properties in several cancers including breast cancer. Recently, we reported FAM83A overexpression in normal breast tissues from women at high risk of breast cancer. We now hypothesize that FAM83A is a key factor in breast cancer initiation.

**Methods:**

Immunohistochemical staining was used to evaluate FAM83A protein levels in both a normal breast tissue microarray (TMA, *N* = 411) and a breast tumor TMA (*N* = 349). EGFR staining and its correlation with FAM83A expression were also assessed. Lentivirus-mediated manipulation of FAM83A expression in primary and hTERT-immortalized breast epithelial cells was employed. Biological and molecular alterations upon FAM83A overexpression/downregulation and FAM83A’s interaction partners were investigated.

**Results:**

TMA analysis revealed a 1.5-fold increase in FAM83A expression level in breast cancer cases as compared with normal breast tissues (*p* < 0.0001). FAM83A protein expression was directly correlated with EGFR level in both normal and breast cancer tissues. In in vitro assays, exogenous expression of FAM83A in either primary or immortalized breast epithelial cells promoted cell viability and proliferation. Additionally, Ingenuity Pathway Analysis (IPA) revealed that FAM83A overexpression in primary cells affected the expression of genes involved in cellular morphology and metabolism. Mass spectrometry analysis identified DDX3X and LAMB3 as potential FAM83A interaction partners in primary cells, while we detected FAM83A interaction with cytoskeleton reorganization factors, including LIMA1, MYH10, PLEC, MYL6 in the immortalized cells.

**Conclusions:**

This study shows that FAM83A promotes metabolic activation in primary breast epithelial cells and cell proliferation in both primary and immortalized cells. These findings support its role in early breast oncogenesis.

**Supplementary Information:**

The online version contains supplementary material available at 10.1186/s40364-022-00353-9.

## Background

Though significant strides have been made in the last several decades in both early diagnosis and treatment of breast cancer, our ability to prevent the disease remains limited by our lack of understanding of the earliest changes of cancer initiation.

Several evidences generated an increasing interest in FAM83 proteins, which have been reported involved in cancer cell signaling and overexpressed in several cancers [1]. FAM83 family (family with sequence similarity 83) consists of 8 genes, FAM83A-H, with a distinct genomic site. The encoded proteins have in common a highly conserved domain of unknown function, the DUF1669 domain, in the N-terminus [2, 3]. The expression of several FAM83 members was reported increased in various cancers, including breast, lung, ovary, cervical, testis, thyroid, bladder, and lymphoid cancers [3, 4].

We recently reported the upregulation of FAM83A in cancer-free breast tissues from women at high risk of developing breast cancer when compared with breasts from average-risk women [5]. FAM83A is a 434-amino acid protein and, in addition to the DUF1669, contains serine-rich and proline-rich domains [6–8]. Several reports point to the role of FAM83A in the regulation of the EGFR pathway in breast cancer cells and in the development of resistance to tyrosine kinase inhibitors (TKIs) [4, 6, 9]. Furthermore*,* overexpression of FAM83A promoted cell migration and invasion through the activation of epithelial-mesenchymal transition (EMT) via PI3K/ATK/Snail signaling [2, 8, 10]. Additionally, FAM83A upregulation was identified in multiple human tumor types, including breast [6, 8, 9], pancreatic [11] and ovarian [1] cancers, as well as lung adenocarcinoma [12]. Moreover, elevated tumor grade and a poor clinical prognosis were observed for patients bearing breast cancer with high expression of FAM83A [13]. Based on the aforementioned evidence, FAM83A may be considered a candidate oncogene. Nevertheless, the role of FAM83A in the early phase of breast cancer development is not fully understood. 

The present study examined the role of FAM83A in the normal breast and in the early phases of breast cancer development. First, FAM83A expression in breast cancer and its correlation with EGFR was evaluated using a large cohort of either normal or cancerous breast tissues. FAM83A overexpression in both primary and immortalized breast epithelial cells promoted metabolic activation and cell proliferation. Finally, we identified differences in FAM83A’s interaction partners between primary and immortalized cells. These findings support the role of FAM83A in breast cancer initiation and, thus, as a potential biomarker for breast cancer susceptibility.

## Methods

### Tissue microarray (TMA) and immunohistochemistry

Normal breast and breast cancer (BC) TMAs were obtained from the Susan G. Komen Tissue Bank at IU Simon Comprehensive Cancer Center (KTB) under Institutional Review Board (IRB) protocol #1011003097 and IUSCCC Tissue Procurement and Distribution Core under IRB protocol #11438, respectively. Demographics and staining data are reported in Additional file 1: Tables S1 and S2. The TMAs were stained with antibodies specific for FAM83A (Protein Tech 20618–1-AP, 1:100) and EGFR (Agilent, K149489, 1:400). Each different antibody underwent a “workup” to determine which steps (or protocol) would best demonstrate the antibody on a particular tissue slide. After de-paraffinizing, TMA sections (4 µm for tumor TMAs and 6 µm for the normal breast TMA) mounted on DAKO slides were subjected to high pH antigen retrieval in the Dako Link PT module. The antibody staining was run on a Dako Link48 with primary antibody for 20 min, Flex-HRP 20 min followed by detection with DAB for 10 min. Three pathologists utilized light microscopy (Leica) to evaluate the immunostaining in each tissue core (score range from negative, slight, moderate, or severe). The slides were imaged using the Aperio Scanscope CS. Computer-assisted morphometric analysis of digital images was performed using the Aperio Image Analysis software included with the Aperio Whole Slide Digital Imaging System [14]. The Positive Pixel Count algorithm was used to quantify the amount of a specific stain present in a scanned slide image. The H-score was calculated using the Aperio TMA software algorithm [14].

### Cell culture and in vitro assays

Primary breast epithelial cells were isolated and cultured as previously described [15]. H-TERT immortalization was obtained upon lentiviral infection of the primary cells with hTERT-expressing lentivirus (Cat# LVP1130 Amsbio, Cambridge MA). H-TERT immortalized cells were then cultured as previously described [16]. Lentiviral infection: Lentivirus containing plasmids encoding shRNAs targeting FAM83A or GFP in pLKO.1 were acquired from Sigma-Aldrich (Sigma Aldrich; sh83A2 TRCN0000168628, sh83A6 TRCN000168368) [33]. GFP-FAM83A or GFP expressing lentivirus particles were obtained from Origene (Lenti ORF particles, FAM83A (mGFP-tagged, transcript 1(CAT#: RC208565L4V) and transcript 2 (Cat# RC219879L4V); Lenti ORF control particles of pLenti-C-mGFP-P2A-Puro, CAT#: PS100093V). Lentivirus at MOI 20 was supplemented with 8 μg/ml polybrene (Santa Cruz) and ViralPlus Transduction Enhancer (Abm, Richmond, BC, Canada, cat: G698, dilution 1:100) before being added to the cell culture. Cells were infected with the virus for 16 h and then washed with PBS1X and cultured with fresh media. Cell proliferation was evaluated by using the bromodeoxyuridine (BrdU) Proliferation Assay and the Sulforhodamine B (SRB) assay. The BrdU cell proliferation assay was conducted according to the manufacturer’s protocol (MilliporeSigma, Burlington, MA). The cells were cultured in 96-well plates at a density of 1 × 10^4^ cells/well for 72 h. Twenty-four h prior, the cells were labeled with BrdU and tagged with the anti-BrdU antibody. Afterwards the substrate was added to the wells, and the colored product was measured using the plate reader (Bio-tek Instruments, USA) at 450 nm. The SRB assay is used for cell density determination, based on the measurement of cellular protein content. 1 × 10^4^ cells/well were cultured in 96-well plates for either 24 h or 96 h. After an incubation period, cell monolayers were fixed with 10% (wt/vol) trichloroacetic acid and stained for 30 min; then, excess dye was removed by washing the cells with 1% (vol/vol) acetic acid. The protein-bound dye was dissolved in 10 mM Tris base solution for optical density, or absorbance, determination at 510 nm using a microplate reader. Experiments included at least four technical duplicates for each condition and were performed three times. The data are shown as percentage of cell-growth calculated using the following equations:

$$\%\mathrm C\mathrm e\mathrm l\mathrm l\;\mathrm g\mathrm r\mathrm o\mathrm w\mathrm t\mathrm h\hspace{0.17em}=\hspace{0.17em}(\mathrm{MeanODsample}/\mathrm{MeanODctr})\hspace{0.17em}\times\hspace{0.17em}100.$$

### RNA isolation and real-time quantitative PCR (qPCR)

Total RNA was extracted from cells using AllPrep DNA/RNA/miRNA kit (Qiagen). Reverse transcription was performed using SuperScript™ IV VILO™ Master Mix (Invitrogen cat#: 11756050) according to the manufacturer’s instructions. qPCR was performed using the TaqMan™ Universal PCR Master Mix (Applied Biosystems, cat# 4304437) and the following TaqMan Gene Expression Assays (Applied Biosystems/Thermo Fisher Scientific, Grand Island, NY): ACTB (Hs99999903_m1), FAM83A (Hs04994801_m1), and NEK2 (Hs00601227_m1). All qPCR reactions were run on the StepOne Plus Real-Time PCR System (Applied Biosystems/Thermo Fisher Scientific) and data analyzed using the StepOne Software v2.3 (Applied Biosystems). Relative quantification was calculated with reference to ACTB and analyzed using the comparative C_T_ method. The qPCR experiments were performed in triplicate.

### Mass spectrometry

Cells were lysed in NP40 lysis buffer (150 mM NaCl, 1% NP-40, 50 mM Tris–Cl pH 8.0) including Protease Inhibitor Cocktail (Sigma P8340) and PhosSTOP (Sigma 4906845001). Cell extracts containing equal quantities of proteins, determined using the BCA Protein Assay kit (Pierce-Thermo Scientific, #23227), were incubated overnight at 4 °C with anti-GFP beads (mGFP/mCFP/mYFP/KillerRed Monoclonal Antibody (OTI2F6), Magnetic beads, TrueMAB, Origene). After 3 × washes with PBS, the samples were then submitted to the IUSM Proteomic Core. A 30 mL Bio-Rad Econoprep column was used to remove resin and bound proteins from the lysate. The column was then washed on with 60 mL TAP lysis buffer. The resin was resuspended in 300μL of 50 mM Ammonium bicarbonate pH 8.0 and transferred to a microcentrifuge tube for onbead digestion with 5μL of Trypsin Gold (0.1 μg/μL) at 37 °C with shaking overnight. The supernatant containing the digested proteins was removed and treated with 20μL of 90% formic acid to inactivate the trypsin. Samples were processed with onbead digestion protocol, cleaned up on spin columns, and ¼ of each was injected on the Eclipse Orbitrap mass spectrometer Thermo Fisher) with FAIMS pro interface [17]. The raw files were searched against the reviewed human UNIPROT database and common contaminants and loaded into Scaffold v5 (Proteomics Software) for easy comparison and viewing.

### Transcriptome analysis

Normal breast tissues from either women prior to their breast cancer diagnosis (susceptible normal, [15]) or age-matched healthy women were obtained from the KTB (Additional file 1: Table S3). Subjects were recruited under a protocol approved by the Indiana University Institutional Review Board (IRB protocol number 1011003097 and 1607623663). Total RNA was extracted from fresh frozen breast tissues using 3 mm zirconium beads (Benchmark Scientific, cat.# D1032-30) and the AllPrep DNA/RNA/miRNA kit (Qiagen) as previously described [5]. Then, cDNA library was prepared using the TruSeq Stranded Total RNA Kit (Illumina, San Diego, CA) and sequenced using Illumina HiSeq4000. Cleaned reads were mapped to Human genome reference sequence GRCh38.p12 with gencode v.28 annotation, using STAR_2.5.2b [18]. Differential Expression Analysis was performed using DESeq2 ver. 1.12.3. The *p-*values from the *t*-test were corrected for multiple testing using Benjamini–Hochberg method.

The dataset is available in the Gene Expression Omnibus (GEO) repository (accession number GSE166044, https://www.ncbi.nlm.nih.gov/geo/query/acc.cgi?acc=GSE166044). Transcriptome profiling of primary breast epithelial cells (*N* = 3) overexpressing either the empty vector (mock, CTR) or FAM83A was performed using Clontech SMARTer Stranded Total RNA-Seq Kit v2 for generating the library, followed by sequencing on NovaSeq v1.5 S4 (200 cycles). Raw data expressed as log2FPKM raw count, and results from differential expression analysis are reported in Additional file 1: Table S4. *T-test* was used to calculate *p*values.

### Data analysis

Ingenuity Pathways Analysis (IPA, Qiagen, Redwood City, CA) was used for canonical pathway and gene network analyses [19]. Publicly available transcriptomic data of microdissected-breast epithelial samples were obtained from GEO with accession number GSE141828 (https://www.ncbi.nlm.nih.gov/geo/query/acc.cgi?acc=GSE141828). Genomic alterations and expression analyses of The Cancer Genome Atlas* (*TCGA, *N* = 3255 for genomic alteration and *N* = 983 for mRNA) and the Molecular Taxonomy of Breast Cancer International Consortium (METABRIC, *N* = 1904 for genetic alteration and *N* = 1405 for mRNA) [20, 21] were performed by interrogating the cBioPortal (https://www.cbioportal.org/) database [22].

### Statistical analysis

Comparisons between groups were done using either Student’s *t*-test or nonparametric Mann–Whitney test on GraphPad Prism 9. Difference between groups is considered significant at *p-*values < 0.05. For the tissue microarray (TMA) data analysis was conducted using SAS software version 9.4 (SAS Institute Inc., Cary, NC). Baseline demographic characteristics were summarized as median (range) for continuous variables and number and percentage for categorical variables. Comparisons between groups for H-score and positivity used a *t*-test to compare results to normal tissue. Correlations were also tested for the patients with both EGFR and FAM83A observations for positivity and H-score using the Wilcoxon test and Pearson’s correlation co-efficient. Finally, FAM83A H-scores and positivity were divided into low and high categories at the score of 29.84702 (H-score) and 0.168319 (positivity) for overall survival (time from surgery to death or censoring). These cutoff values were determined by using the maximum chi-square value for all score values between the 25th and 75th percentile (http://www.pharmasug.org/proceedings/2012/SP/PharmaSUG-2012-SP12.pdf). Kaplan–Meier analysis was used to analyze the data and used the log-rank *p*-value to compare the two groups.

## Results

### FAM83A protein upregulation in breast cancer (BC)

FAM83A was first discovered for its ability to induce cell transformation in breast epithelial cells [6]. The *FAM83A* locus is located on chromosome 8q24.13, which is frequently amplified in several human cancers [23]. Analysis of FAM83A genomic aberrations in the METABRIC TCGA dataset revealed FAM83A locus amplification in 23% and 15% of BC, respectively (Additional file 2: Supplementary Fig. 1A). Overexpression of FAM83A protein level in Her2 positive (HER2 +) BC was previously reported [8], and FAM83A mRNA upregulation in HER2 + BC was also detected in TCGA and METABRIC databases (Additional file 2: Supplementary Fig. 1B-C).

**Fig. 1:**
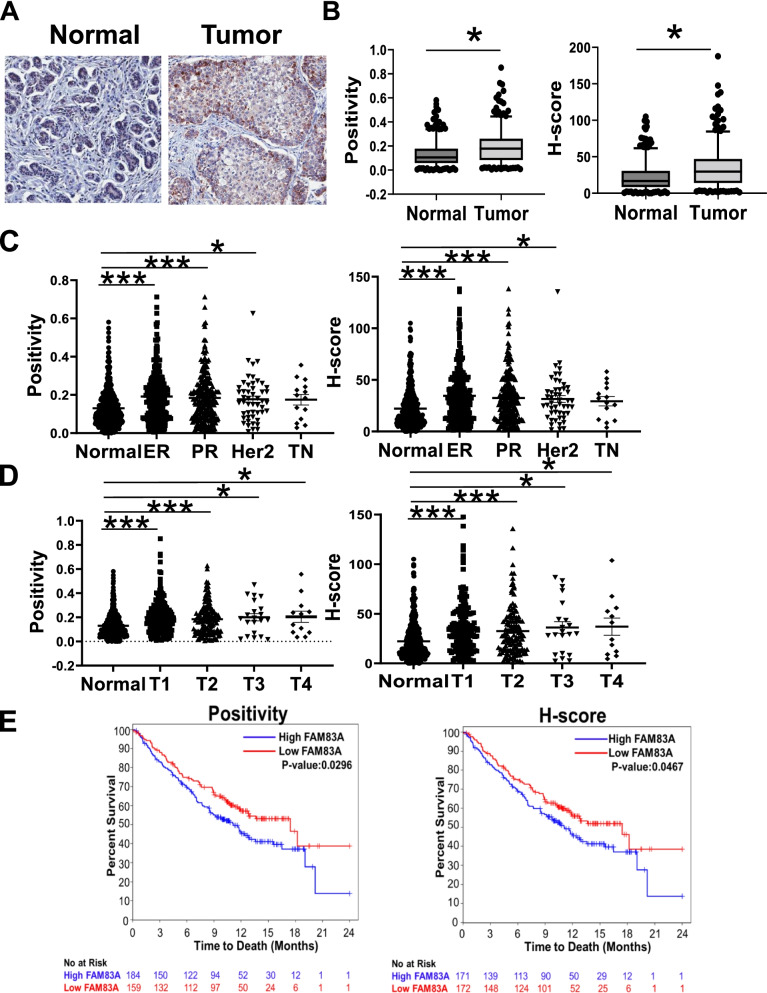
FAM83A expression in breast tumors. **A** Representative images at 20 X magnification and **B** quantification of FAM83A staining of either normal or tumor breast tissue sections. Data are shown as box and whisker at 5th-95th percentile. **C** FAM83A staining quantification in different breast cancer hormone receptor-based subtypes or **D** tumor stage (T1-T4) as compared with normal breast. Each dot represents a single subject data point. Data are shown as average ± standard error of the mean. **E** Kaplan–Meier survival curves of breast cancer patients based on FAM83A expression status for both positivity and H-score analyses (blue lines indicate patients with high FAM83A level; red lines indicate patients with low FAM83A level). ER: estrogen receptor, PR: progesterone receptor, Her2: HER2 gene amplification, TN: triple negative. *p < 0.05.**p < 0.001, ***p < 0.0001

Here, we examined the expression of FAM83A in both normal breast tissues (*N* = 411) and breast cancer (BC) cases (*N* = 349) (Fig. 1A and B, Additional file 1: Tables S1, S2 and S5). Immunohistochemical analysis revealed a 1.5-fold increase in FAM83A protein levels in breast tumors as compared with the normal breasts (*p* = 5E-13). Furthermore, we observed an increase in FAM83A protein in estrogen receptor positive (ER + ; fold change (fc):1.5 for positivity, *p* = 3E-11 and fc:1.7 for H-score, *p* = 1E-11) and progesterone receptor positive (PR + ; fc:1.6 for positivity, *p* = 1E-08 and fc:1.7 for H-score, *p* = 1E-08) BC as well as in HER2 + BC (fc:1.6 for positivity, *p* = 0.002 and fc:1.6 for H-score, *p* = 0.002) as compared with normal breasts (Fig. 1C and Additional file 1: Table S5). No significant difference in FAM83A expression level between normal tissue and triple negative BC (TNBC) was observed, probably due to the limited size of the TNBC cohort (*N *= 19). FAM83A was overexpressed in early stages of BC (T1, *p* = 1.8E-12 and T2, *p* = 6E-07 for both positivity and H-score analyses) more than in the later stages (T3: *p* = 0.001 and T4: *p* = 0.01 for positivity; T3: *p* = 0.001 and T4: *p* = 0.008 for H-score, Fig. 1D). No differences in FAM83A levels in relation with lymph node positivity (N) and metastatic disease (M) were observed (Additional file 2: Supplementary Fig. 1D and E). Moreover, Kaplan–Meier curves indicated that the overall survival was significantly worse in high FAM83A expression BC cases as compared with the cases with low FAM83A expression (*p* = 0.02 for positivity data; *p* = 0.04 for H-score data; Fig. 1E).

### FAM83A expression increased in early phase of BC development

**Fig. 2:**
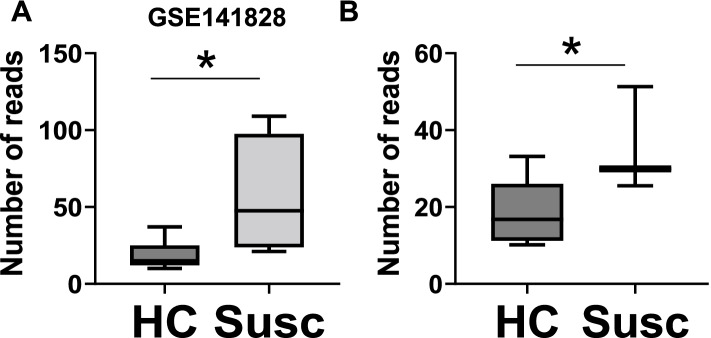
FAM83A expression in breast prior to cancer diagnosis. **A** FAM83A expression in microdissected-epithelial breast tissues from either women prior their breast cancer diagnosis (Susc) or healthy women (healthy controls, HC). Data were retrieved from GEO, GSE141828. **B** FAM83A expression in breast tissues from either women prior their breast cancer diagnosis (Susc) or healthy women (HC). *p < 0.05

We recently reported FAM83A upregulation in normal breasts from women at high risk of developing breast cancer as compared with breasts from average-risk women, according to the Tyrer-Cuzick risk estimation model [5]. The correlation of FAM83A protein level with the estimated BC risk score was confirmed by using an independent immunostaining of our larger sample cohort (Additional file 2: Supplementary Fig. 2A). Then, we examined FAM83A expression in histologically normal breast tissues from either healthy women (healthy control, HC) or women who had later a diagnosis of BC (susceptible normal, Susc) retrieved from the GSE141828 dataset (Fig. 2) [15]. Only data points with > 10 reads were included in the analysis. FAM83A showed a 3.9-fold increased expression in Susc (*N* = 4) as compared with the HC (*N* = 8) (*p* = 0.02) (Fig. 2A). To confirm these data, we performed a transcriptomic analysis of whole breast tissue from an independent cohort of either HC or Susc (Additional file 1: Table S3**)**. FAM83A expression showed a 1.9-fold increase in Susc (*N* = 3) breasts as compared with HC samples (*N* = 8) (*p* = 0.03; Fig. 2B), suggesting an upregulation of FAM83A in the early phase of breast cancer development. To verify our observation, we analyzed the dataset generated by Aran et al. [24] including transcriptome profiling of normal breast (from the GTEx database), normal adjacent to tumor (NAT, from the TCGA database) and breast tumors (from TCGA). FAM83A expression increased in the NAT compared with the normal breast (logfc: 3.96, *p* = 9.9E-20), and also compared with the tumor (logfc: 2.69, *p* = 1.0E-18) [24]. Next, we examined FAM83A mRNA expression in primary epithelial cells generated from breast tissue biopsies of healthy women, and isogenic hTERT-immortalized and hTERT/RAS-transformed cells described in Kumar et al. [25]. FAM83A expression increased 6.2-fold in the immortalized cells (*p* = 0.01), while only 4.3-fold in the transformed cells (*p* = 0.03) (Additional file 2: Supplementary Fig. 2B). Both our findings and the data from Aran et al. suggest that FAM83A is specifically activated in the early/intermediate phase of BC development but may have a limited impact on the later phases of cancer progression.

### FAM83A expression correlates with EGFR level

**Fig. 3:**
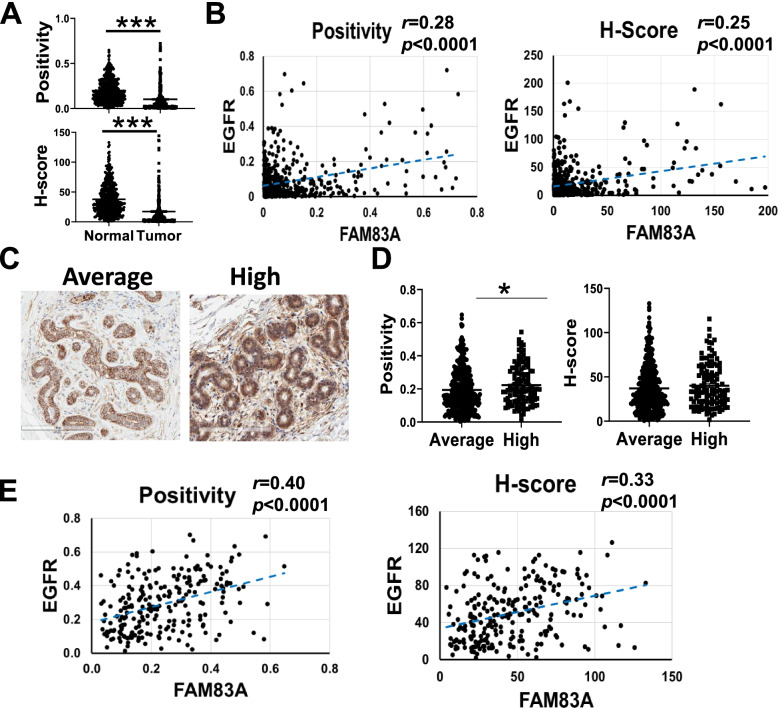
EGFR and FAM83A correlation in cancerous and normal breast. **A** EGFR staining quantification in normal breast (N) and Tumor (T) tissues. Staining quantification is expressed as positivity and H-score. Data are shown as mean ± standard error of the mean. **B** Pearson’s correlation between FAM83A and EGFR level in breast tumors. **C** Representative images of EGFR staining of normal breast tissues from women at either average or high risk for breast cancer. 20X magnification is shown. **D** Quantification of EGFR staining of normal breast expressed as positivity and H-score. **E** Pearson’s correlation between FAM83A and EGFR level in normal breast tissues. *p < 0.05, ***p < 0.0001

FAM83A protein has been reported as a possible key regulator in the EGFR pathway in BC cells, leading to the development of resistance to tyrosine kinase inhibitors (TKIs) [6, 8]. A recent report revealed a direct correlation between EGFR and FAM83A expression in NSCLC [12]. We examined EGFR expression in both tumor and normal breast TMA and its correlation with FAM83A protein level (Fig. 3). As previously reported [26], and in accordance with what was observed in the METABRIC and TCGA cancer datasets (Additional file 2: Supplementary Fig. 3), EGFR expression is downregulated in tumors as compared with the normal breasts (2.4-fold reduction, *p* < 0.0001 for positivity, 2.6-fold reduction, *p* < 0.0001 for H-score; Fig. 3A). Nevertheless, EGFR expression in BC is directly correlated with FAM83A levels (*r* = 0.28 for positivity and *r* = 0.25 for H-score, both at *p* < 0.0001) (Fig. 3B). A direct correlation between FAM83A and EGFR (Spearman rho: 0.24) was also detected in the epithelial fraction from the single-cell transcriptome profiling of BC tissue performed by Wu et al*.* (Additional file 2: Supplementary Fig. 4A, [27]). When separating the data based on BC subtypes, the direct correlation was most evident in TNBC cases. In light of this data, we analyzed the FAM83A and EGFR immunostaining in the different BC subtypes present in our TMA and found a significant EGFR-FAM83A correlation in ER + BC (*N* = 176; *r* = 0.21, *p* = 0.004 for positivity and *r* = 0.15, *p* = 0.04 for H-score). However, no significant correlation was detected in HER2 and TNBC, mostly due to the limited size of these cohorts (HER2 N= 37 and TNBC N= 9; Additional file 2: Supplementary Fig. 4B).

A 1.2-fold overexpression of EGFR protein was also detected in tissues from women at high risk for BC as compared to the average-risk group (Fig. 3C and D). However, the difference in EGFR expression between the two groups was significant only for the positivity analysis (*p* = 0.01) and not for the H-score analysis (*p* = 0.11). Pearson’s correlation analysis revealed a direct correlation between the EGFR and FAM83A expression levels in normal breast tissues (*r* = 0.40 for positivity and *r* = 0.33 for H-score, both at *p* < 0.0001) (Fig. 3E). Data suggest that FAM83A may be involved in the EGFR signaling in both normal and cancer cells.

### FAM83A overexpression promotes metabolic activation and cell proliferation of primary breast epithelial cells

**Fig. 4:**
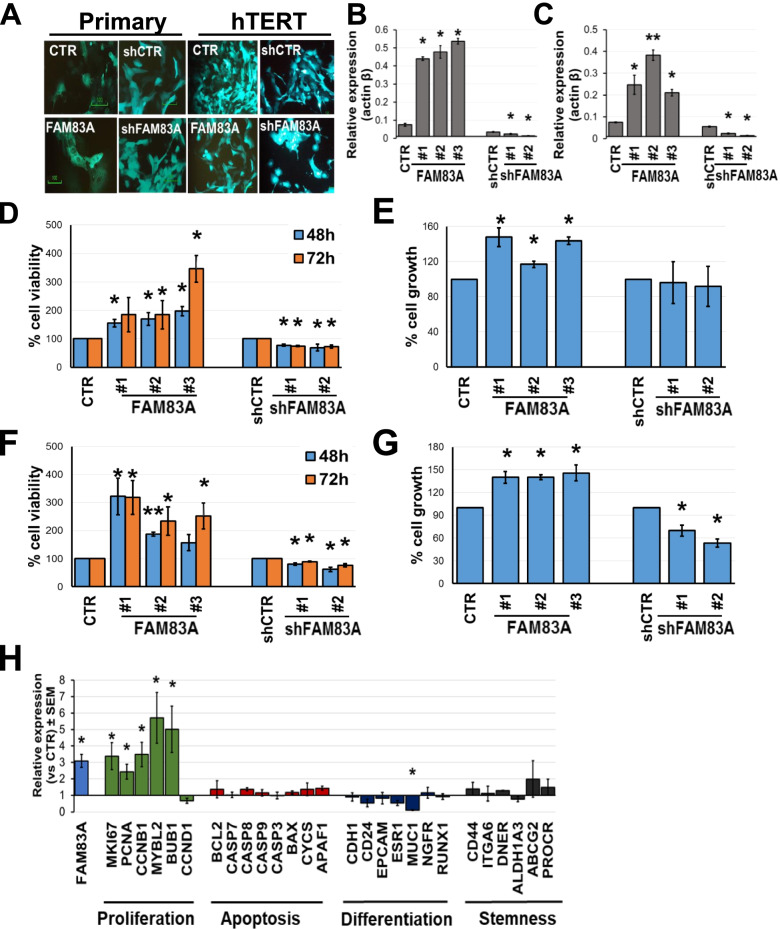
FAM83A overexpression promotes cell proliferation. **A** Representative images of either mGFP-positive primary epithelial cells (*N* = 3) or paired hTERT-immortalized cells after lentiviral infection to express either control mock (CTR), FAM83A (FAM83A), pLKO control (shCTR), or shRNA for FAM83A (shFAM83A). 20 × magnification images are shown and mGFP is in green. Level of FAM83A expression in either primary (**B**) or immortalized (**C**) cells infected with either control, FAM83A-overexpression, or shFAM83A lentivirus particles. Cell viability and cell proliferation of primary (**D** and **E**) or immortalized (**F** and **G**) cells measured with SRB assay or BrdU assay, respectively. Data are shown as percentage of either cell viability or cell growth of the FAM83A overexpressing/downregulating cells versus the control cells. **H** Expression of cell proliferation-, apoptosis-, differentiation-, and stemness-related genes in primary epithelial cells overexpressing FAM83A (*n* = 3) as compared to mock-expressing cells (*n* = 3). Data are shown as mean ± standard error of the mean (SEM). *p < 0.05, **p < 0.001

To investigate the functional role of FAM83A in normal breasts as well as in cell transformation, we employed mGFP tagged-lentivirus infection to manipulate FAM83A expression in both primary and hTERT-immortalized epithelial cells (Fig. 4A, B and C). Primary breast epithelial cells were isolated as previously described [15] from the cryopreserved breast tissue cores of average-risk women, and then infected with hTERT-expressing lentivirus. We next sought to determine whether FAM83A overexpression or loss impacted proliferation capacity or survival. First, cell viability and proliferation were monitored using Sulforhodamine B and bromodeoxyuridine (BrdU) incorporation assays, respectively (Fig. 4). Compared with the control cells, both cell viability (up to 90% increase at 48 h and 3.4-fold at 72 h, *p* > 0.05) and proliferation rate (up to 47% increase, *p* < 0.05) of FAM83A-overexpressing primary epithelial cells were significantly increased (Fig. 4D and E). However, after downregulating the expression of FAM83A in primary epithelial cells, a reduction in cell viability was detected (22% reduction, *p* = 0.003, for clone#1 and 31% reduction, *p* = 0.04, for clone#2 at 48 h; 25% reduction, *p* = 0.03, for clone#1 and 26% reduction, *p* = 0.04, for clone#2 at 72 h), whereas no change in cell proliferation was observed (Fig. 4D and E). In the immortalized cells, the overexpression of FAM83A induced a significant increase in both cell viability and proliferation as compared with the control cells (*p* < 0.05, Fig. 4F and G). In contrast, when the expression of FAM83A was downregulated, the cell viability and proliferation rate of immortalized breast epithelial cells significantly decreased compared with those of control cells (*p* < 0.05; Fig. 4F and G).

Next, we assessed how FAM83A overexpression in primary epithelial cells impacted the expression of survival-, cell differentiation-, and proliferation-related genes (Fig. 4H). Because of the limited growth rate of the shFAM83A-primary cells and limited RNA yield, the transcriptome profiling generated a small number of reads (< 15,000), below the appropriate threshold, and therefore the data were not included in any further analysis. The expression of key markers associated with proliferation (MKI67, PCNA, CCNB1, MYBL2, BUB1, CCND1), apoptosis (BCL2, CASP7, CASP8, CASP9, CASP3, BAX, APAF1), differentiation (CDH1, CD24, EPCAM, ESR1, NGFR, RUNX1), and breast cancer stemness (CD44, ITGA6, DNER, ALDH1A3, ABCG2, PROCR) was assessed. FAM83A overexpression in primary epithelial cells induced the upregulation of several proliferation markers (*p* < 0.05); however, most cell differentiation genes were unaffected by the FAM83A overexpression, except for MUC1. This gene, a marker of luminal epithelial cells, showed a significant downregulation in FAM83A- overexpressing as compared with the mock-expressing primary cells (fc:0.1; *p* = 0.03). In normal cells MUC1 creates a physical barrier and protects the underlying epithelia from pH changes, pollutants, and microbes [28]. No change in the expression of apoptosis- and stemness-related genes was detected. The data suggest that, at this early stage of cell transformation, the proliferating epithelial cells overexpressing FAM83A do not show yet signs of cell plasticity or dedifferentiation.

**Fig. 5:**
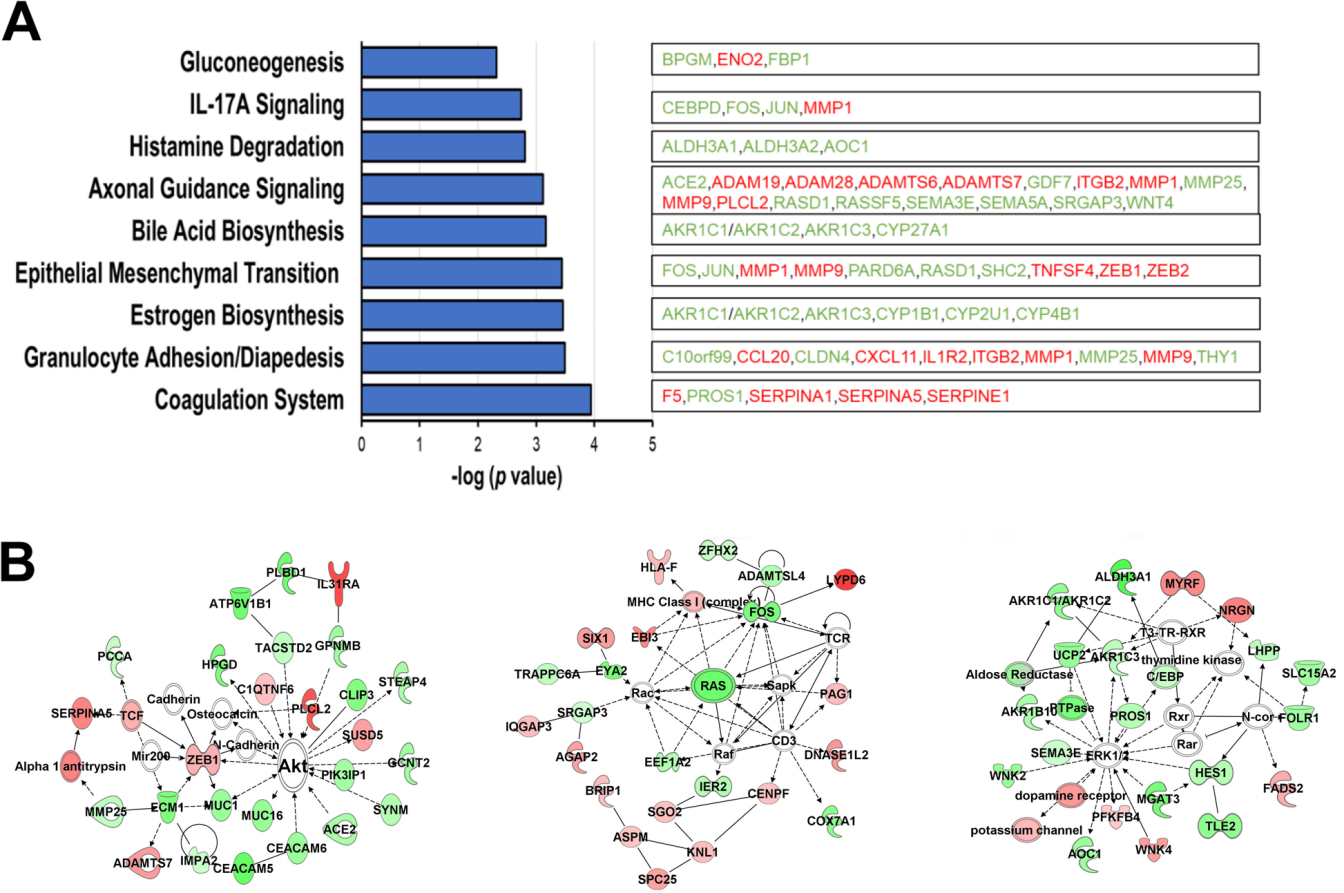
FAM83A overexpression induced metabolic pathway activation in primary epithelial cells. **A** Ingenuity pathway analysis (IPA) revealed the canonical pathways linked with the genes differentially expressed between FAM83A-overexpressing and control cells. Downregulated genes are in green and upregulated genes are in red. **B** Molecular networks linking the differentially expressed genes were obtained using IPA. Upregulated genes are shown in red while downregulated genes are in green and connecting molecules are white. The three networks shown in the figure include: Cancer and endocrine system disorders (left); Cancer and cell morphology (center); and Carbohydrate metabolism (right)

Transcriptome profiling of either FAM83A- or mock-overexpressing primary cells was performed followed by differential expression analysis using DeSeq2 (Additional file 1: Table S4). Upon FAM83A overexpression in primary epithelial cells, 26 genes transcripts were downregulated, whereas 179 genes were upregulated compared with the control (mock-expressing) cells at a fc ≥ :2 and *p* < 0.05. The majority of the genes affected by FAM83A overexpression were involved in cell adhesion (*p* = 0.0003), epithelial mesenchymal transition (*p* = 0.0003), metabolism (*p* = 0.005) and estrogen biosynthesis (*p* = 0.0003) (Fig. 5A). The latter included downregulated genes only. The genes with the highest upregulation level and lowest *p* value included the metabolic factors ACOT4 (fc: 1.9, *p* = 0.001), CA9 (fc:9.05, *p* = 0.001), and PDK1 (fc:2.2, *p* = 0.004), and cell adhesion-related molecules PCDH12 (fc:9.47, *p* = 0.001) and CAVIN3 (fc:3.93, *p* = 0.002). Molecular networks including the differentially expressed genes between FAM83A-overexpression and controls, and with a score > 30 were the following: Cancer and endocrine system disorders (score 45), Cancer and cell morphology (score 38), and Carbohydrate metabolism (score 31) (Fig. 5B).

### FAM83A shows unique interaction partners in primary and immortalized epithelial cells

To determine the mechanism of action of FAM83A in epithelial cells, protein interaction partners were also investigated. GFP-tagged FAM83A was overexpressed in either primary or hTERT-immortalized breast epithelial cells. Affinity purification with anti-GFP antibody combined with mass spectrometry (AP-MS) was used for protein–protein interactions mapping [29]. Only targets associated with more than 20 peptides detected in the FAM83A overexpressing samples were considered for the analysis. Moreover, because a large number of nonspecific interactors, or contaminants, are co-purified with bait proteins and identified by MS, previously identified contaminants were selected and removed from the analysis [30, 31]. In the primary epithelial cells two proteins were found interacting with FAM83A: the helicase DDX3X and laminin subunit beta-3, LAMB3 (Table 1 and Additional file 1: Table S6). While the first is involved in post-transcriptional regulation [32], LAMB3 plays a role in cell growth and cell adhesion [33]. In the hTERT-immortalized cells, FAM83A interacts with four molecules including LIMA1, PLEC, MYL6 and MYH10, involved in cytoskeleton reorganization.

**Table 1:** FAM83A protein–protein interaction partners

Name	Description	Accession Number	Molecular Weight	FC^a^	*p* value^##^
**Primary breast epithelial cells**					
DDX3X	ATP-dependent RNA helicase	O00571	73 kDa	2.60	0.002
LAMB3	Laminin subunit beta-3	Q13751	130 kDa	12.67	0.040
**hTERT-immortalized breast epithelial cells**					
LIMA1	LIM domain and actin-binding protein 1	Q9UHB6	85 kDa	3.07	0.013
PLEC	Plectin	Q15149	532 kDa	7.08	0.017
MYL6	Myosin light polypeptide 6	P60660	17 kDa	1.88	0.036
MYH10	Myosin-10	P35580	229 kDa	3.39	0.037

^a^*FC* Fold change, FAM83A-overexpressing vs control-expressing samples; ^##^two-tailed Student *t*-test

## Discussion

FAM83A is overexpressed in a variety of human tumors including lung, breast, testicular, and bladder cancers, suggesting that FAM83A may play a role in the development of cancer [2, 34, 35]. Here, we investigated FAM83A association with breast cancer (BC) susceptibility by examining its expression in normal and BC tissues and evaluating its functional role in the normal breast epithelial cells. We explored indeed both transcriptome alterations downstream FAM83A overexpression/downregulation, and FAM83A interaction partners in both primary and immortalized breast epithelial cells.

We recently reported the upregulation of FAM83A in the breast tissue from women at high risk of developing BC (Tyrer-Cuzick lifetime risk score ≥ 20%), suggesting its potential role in BC susceptibility [5]. Notably, in this study we observed FAM83A upregulation not only in BC cases but also in breast tissue prior to BC diagnosis (pre-diagnosis or “susceptible”) compared to age-matched healthy controls. By using data from the GTEx and TCGA databases, Aran et al. reported a transcriptome profiling of the normal breasts, tumor, and histologically normal breast tissue adjacent to the tumor (NAT). The authors described the NAT as an intermediate state, representing not just a gradient between tumor and healthy tissue, but a distinct tissue phenotype. They identified 82 genes that were upregulated in NAT compared with both healthy tissue and tumor. Among those, FAM83A expression increased in the NAT compared with either the normal breast (logFoldChange: 3.96, *p* = 9.9E-20) or the tumor tissue (logFoldChange: 2.69, *p* = 1.0E-18) [24]. Together the data suggest that FAM83A may have an important role in the early phase of breast cancer development rather than in late phase when the tumor mass is well-defined and oncogenic pathways prevail.

In regard to FAM83A expression in BC, previous transcriptomic and proteomic studies revealed that FAM83A is upregulated in HER2 + BCs, including those that are resistant to the HER2-targeted therapy trastuzumab [8, 36]. HER2 is amplified in about 30% of all breast cancer patients and is a member of the same receptor tyrosine kinase family as EGFR, the ErbB family. In our immunohistochemical analysis, including 411 normal and 349 BC cases, we detected FAM83A overexpression in BC as compared with normal breasts and an equal expression of FAM83A among ER, PR, and HER2 + BC. However, FAM83A was not significantly overexpressed in triple negative BC (TNBC) as compared with normal breasts, probably due to the limited size of this cohort (*N* = 19). Moreover, FAM83A protein expression increased mostly in the early BC stages (T1 and T2) rather than in later stages (T3 and T4). Nevertheless, as previously reported [13], Kaplan–Meier survival curves from the cancer cohort indicated that high FAM83A levels correlated with shortened survival time and poor prognosis in BC patients. Though initially this may seem to contradict our hypothesis that FAM83A is critical to the earliest phases of BC initiation, and less so to cancer local progression and invasion, we propose at least two explanations for FAM83A’s resurgent expression in advanced disease: [1] FAM83A’s involvement in treatment resistance [6]; and [2], as observed in other tumor types [10], FAM83A’s role in the late phase of breast tumorigenesis, potentially metastatic disease, which has not been investigated here. Our results provide strong evidence for a critical role of FAM83A in early phase of BC development, and, as such, it should be explored as a therapeutic target.

The ErbB family includes four members: ErbB1/EGFR, ErbB2/HER2/Neu, ErbB3/HER3, and ErbB4/HER4. The ErbB signaling regulates normal cell growth and proliferation through the activation of several pathways such as RAS/MAPK and PI3K/AKT/mTOR pathways, STAT3, and Phospholipase D [37]. Precision therapies aimed at disrupting ErbB RTKs (erlotinib, gefitinib, cetuximab, lapatinib, trastuzumab, pertuzumab), and downstream pathway (RAS-, RAF-, MEK-, PI3K/AKT/mTOR pathway inhibitors) are currently approved for patient use or are being evaluated in several clinical trials [38–41]. Previous studies report that BC cell lines with higher FAM83A expression (T47D, MCF7, MDA-MB361, MDA-MB468, and MDA-MB231) were more resistant to EGFR-TKI than cell lines with moderate expression (SKBR and T4-2) [6]. In our analysis, we found a direct correlation between FAM83A and EGFR protein levels stronger in normal (*r* = 0.4, *p* < 0.0001) than in BC (*r* = 0.2, *p* < 0.0001) samples suggesting that FAM83A may be involved in the EGFR-driven proliferative pathway in the normal breast and early phase of BC development rather than only in advanced BC as previously described [6].

FAM83A was reported to drive transformation and resistance to EGFR-TKIs in HMEC cells [6]. FAM83A overexpression in vitro enhanced cell proliferation and invasiveness of various cancer cell lines, including human lung [34, 42], pancreatic [11] and BC [6]. Depletion of FAM83A by shRNA in BC cell lines HMT-3522 T4-2 and MDA-MB-468 decreased invasiveness, proliferation rate, clonogenic potential, and tumor growth in immunocompromised mice [2, 6]. However, FAM83A knockdown in human cervical cancer cell lines HeLa and CaSki promoted tumor growth and invasion [43]. In our study, FAM83A overexpression in primary breast epithelial cells induced both cell viability and proliferation. Furthermore, as previously reported [6], FAM83A overexpression in immortalized breast epithelial cells also promoted both cell viability and proliferation, which decreased instead after FAM83A depletion. However, while FAM83A downregulation in primary epithelial cells decreased the SRB staining (used to measure cell viability), probably reflecting a reduction in cell metabolism, no effect on BrdU incorporation (used to measure cell proliferation) was observed. The findings suggest that FAM83A affects cell growth of primary breast epithelial cells, although its pro-oncogenic role is more evident in the immortalized cells. Furthermore, the transcriptome analysis of FAM83A overexpressing primary cells confirmed the influence of FAM83A on cell metabolism and pro-oncogenic signaling pathways. This is the first study describing the effect of FAM83A overexpression in primary breast epithelial cells.

Finally, we further investigated FAM83A’s mechanism of action in both primary and immortalized breast epithelial cells by searching for potential interaction partners. Among the eight FAM83 family genes (FAM83A-H), FAM83A protein is the smallest one (434 amino acids) and contains DUF1669, serine-rich, and proline-rich domains [6]. The proline-rich domains include a conserved PxxP motif, which interacts with Src homology 3 domain-containing proteins [44]. Recently it was found that the DUF1669 domain of FAM83 family proteins anchored casein kinase 1 isoforms which are implicated in the regulation of many cellular processes [7]. Co-immunoprecipitation experiments revealed that FAM83A interacts with both c-Raf and the p85 regulatory subunit of PI3K following stimulation with EGF [6]. Moreover, FAM83A can be phosphorylated upon EGFR activation, and FAM83A phosphorylation may regulate signaling complex formation and FAM83A-mediated transformation. In our investigation of FAM83A’s role in primary breast epithelial cells, upon performing an affinity mass spectrometry experiment, we observed that in both primary and immortalized cells FAM83A binds actin filaments related protein, and KEGG pathway investigation associates the identified targets with tight junction (MYHL6, MYH10) and focal adhesion/ECM- receptor interaction (LAMB3). Lee et al*.* reported that FAM83A depletion caused actin stress fibers to become primarily cortical leading to reduced invasiveness [6]. Notably, another FAM83A interaction partner in primary cells is DDX3X, a molecule of interest in cancer biology for its involvement in cell cycle, apoptosis, and cell migration through regulation of transcription, mRNA maturation, mRNA export, and translation [32].

Although this is the first report using a large cohort of either normal or cancerous breast tissues to investigate FAM83A’s protein expression and analyzing FAM83A’s functional role in primary breast epithelial cells, the study bears several limitations. Because 92% of the tumor cohort consisted of ductal/lobular invasive disease, our analysis lacked the FAM83A staining data on the earlier premalignant lesions. While we tried to address this point by analyzing the susceptible normal breast cohort (Fig. 2), the analysis of early phase of BC in the PreCancer Atlas may provide better clues on the significance on FAM83A in BC progression [45]. The study lacks the in vivo validation of the growth-promoting feature of FAM83A overexpressing cells. While implantation of immortalized and transformed breast epithelial cells into the mammary fat pad of NSG mice has been successful performed [46, 47], the xenograft model of human primary breast epithelial cells is not yet optimized. Furthermore, while new interaction partners for FAM83A in both primary and immortalized cells were identified, the functional impact of such interaction requires further investigation. Previous studies demonstrated that the elevated FAM83A expression observed in numerous tumor types could be due to genomic amplification of 8q24, which also contains the oncogene Myc [11]. It would be interesting to evaluate the correlation between FAM83A level and 8q24 amplification in our cohort and examine other potential mechanisms responsible for FAM83A induction.

## Conclusions

In summary, our study elucidates the critical role of FAM83A in breast cancer initiation. When compared with its level in normal breast, FAM83A shows an increased expression in both breast tumors and, to a greater extent, in early phase of BC development, as suggested by our data on susceptible normal breast and Aran et al.’s data on NAT [24]. Moreover, FAM83A overexpression in primary cells induced metabolic activation and cell proliferation, two hallmarks of cancer initiation. Our study also demonstrates the importance of examining the normal breast tissue to decipher the molecular aberrations occurring in early breast cancer development. Understanding the earliest changes in malignant transformation will be critical to eventually preventing this disease altogether.

## Supplementary Information

**Additional file 1.** It includes subjects demographics and raw data in form of tables.

**Additional file 2.** It includes additional data related to the main findings shown in the main figures.

## Data Availability

The dataset including transcriptome profiling of microdissected breast epithelium form either susceptible normal breast biopsies or healthy controls was retrieved from the Gene Expression Omnibus (GEO) repository, access number GSE141828 (https://www.ncbi.nlm.nih.gov/geo/query/acc.cgi?acc=GSE141828). Transcriptome profiling of susceptible breast samples and age-matched healthy control is available in GEO (accession number GSE166044, https://www.ncbi.nlm.nih.gov/geo/query/acc.cgi?acc=GSE166044). The transcriptome data (raw counts) for the FAM83A- or CTR-overexpressing primary cells are included in Additional file 1: Table S5. The mass spectrometry data (raw counts) for the FAM83A- or CTR-overexpressing primary and hTERT immortalized cells are included in Additional file 1: Table S6.

## Abbreviations

BC: Breast cancer
KTB: Susan G. Komen Tissue bank at IU Simon Comprehensive Cancer Center
TMA: Tissue MicroArray
IPA: Ingenuity pathway analysis
Susc: Susceptible
HC: Healthy Control
