# Supplementary material for: FAM83A is a potential biomarker for breast cancer initiation
Source: Biomark Res. 2022 Feb 19;10:8. doi: 10.1186/s40364-022-00353-9 (PMC8858535; doi:10.1186/s40364-022-00353-9)
Supplement: Supplementary file 2 — Additional file 2. It includes additional data related to the main findings shown in the main figures. [file 40364_2022_353_MOESM2_ESM.pdf]

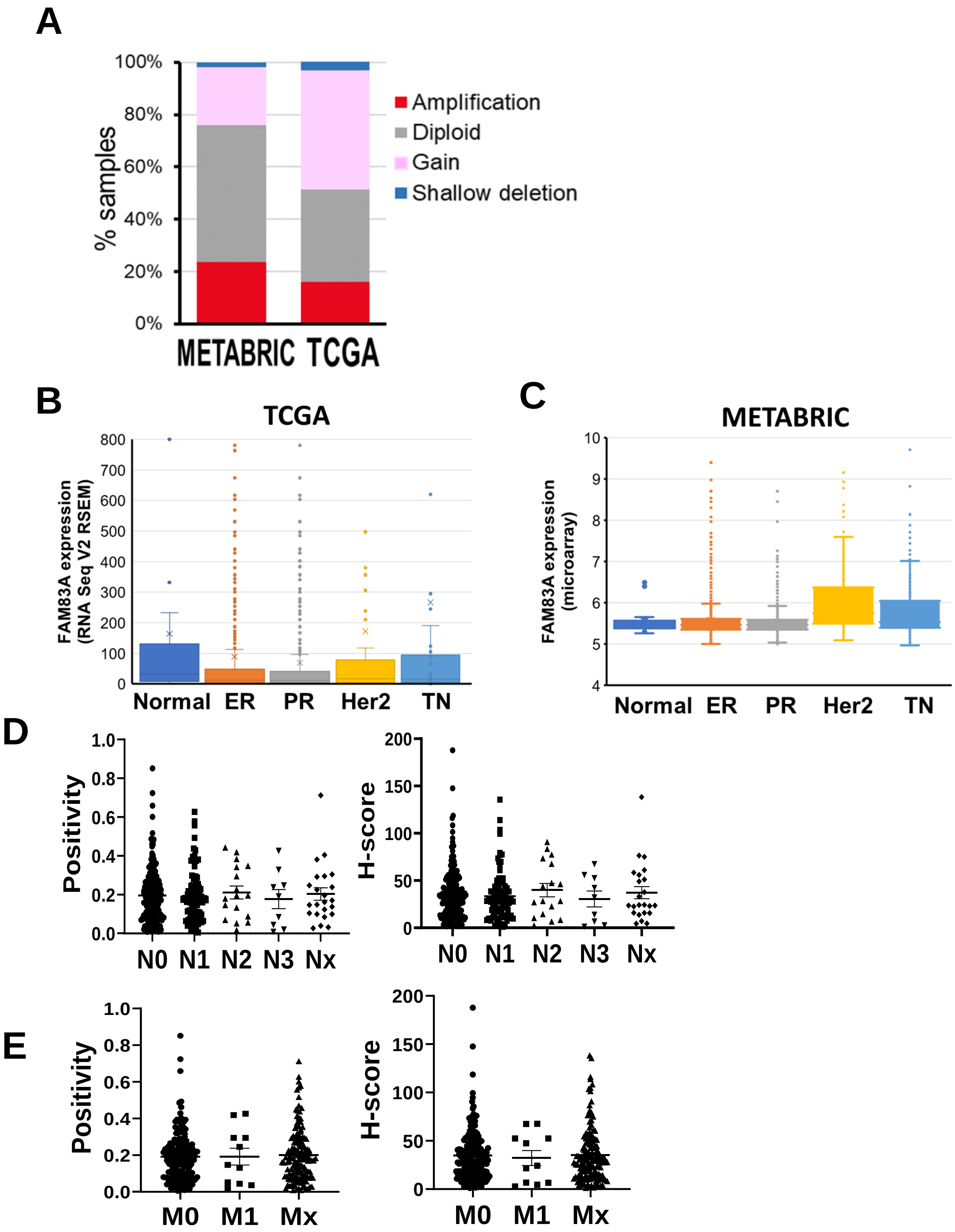


**Supplementary Figure 1: FAM83A expression in breast cancer data base. A**) FAM83A genomic aberrations in breast cancer from either METABRIC (N=1904) or TCGA (N=3255) dataset. Data of FAM83A mRNA expression in normal and breast cancer subtypes (based on hormone receptor expression) are retrieved from either the (**B**) TCGA or (**C**) METABRIC dataset using cBioportal database. FAM83A expression in breast cancer cases divided by either (**D**) lymph nodes positivity (N) or (**E**) metastatic disease (M) staging. Data from both positivity (left panel) and H-score (right panel) are shown. Each dot represent a data point. Data are shown as mean ± standard error of the mean.

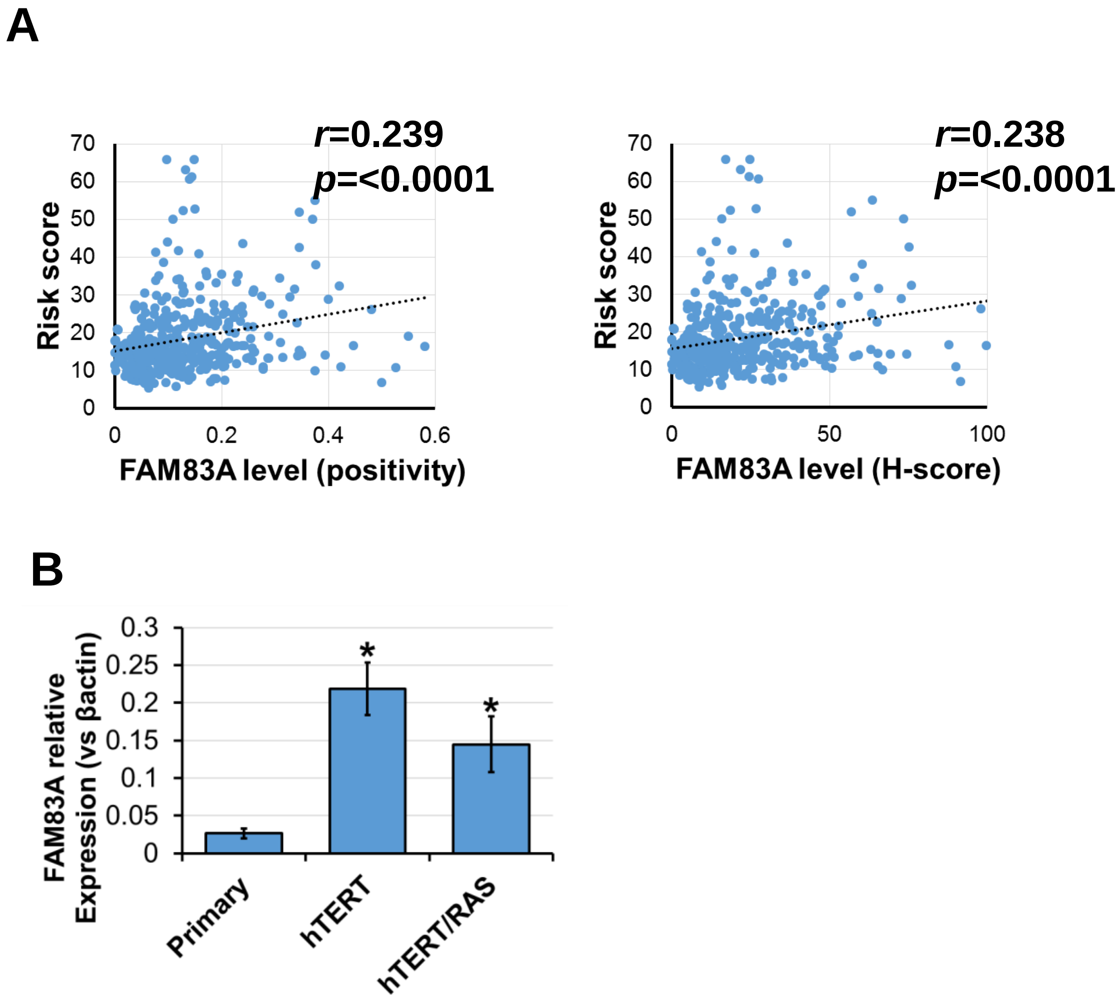


**Supplementary Figure 2: Correlation of FAM83A expression with breast cancer risk and progress. A**) Pearson's correlation analysis revealed a direct link between FAM83A expression in normal breasts (N=408) and the Tyrer-Cuzick breast cancer risk score. Data are shown as positivity analysis (left panel) and H-score analysis (right panel). **B**) FAM83A expression in either primary, hTERT-immortalized, or hTERT/RAS-transformed breast epithelial cells evaluated by using qPCR. *:$p<0.05$

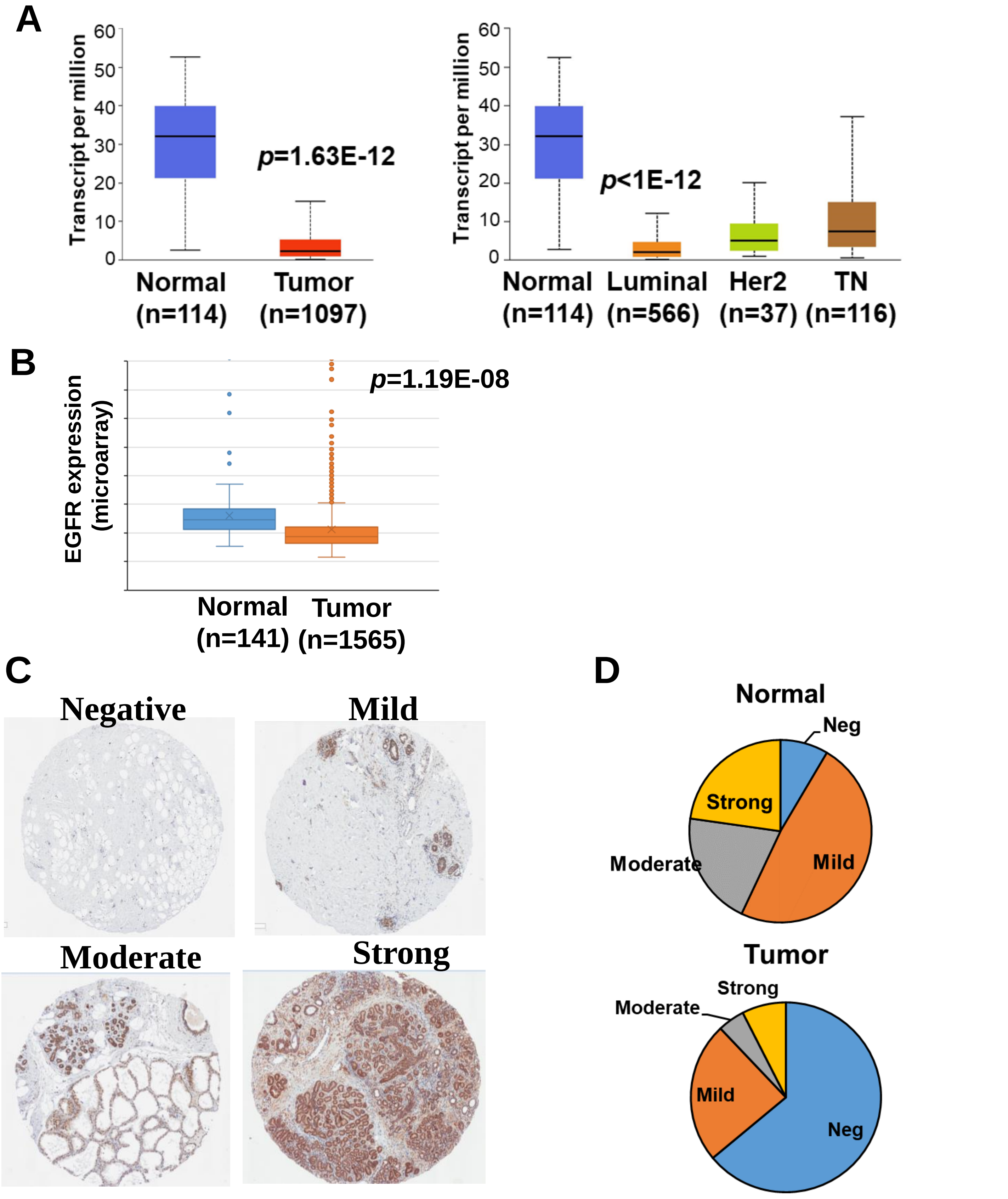


**Supplementary Figure 3**: **EGFR expression in tumor and normal breast.** EGFR expression was surveyed in the (**A**) TCGA and (**B**) METABRIC datasets. **C**) Representative images at 5.6 magnification of different EGFR staining intensities in the tumor TMA: Strong , Moderate , Mild and Negative. **D**) Pie chart showing the proportion of each staining intensity in either the normal breast TMA or the breast tumor TMA.

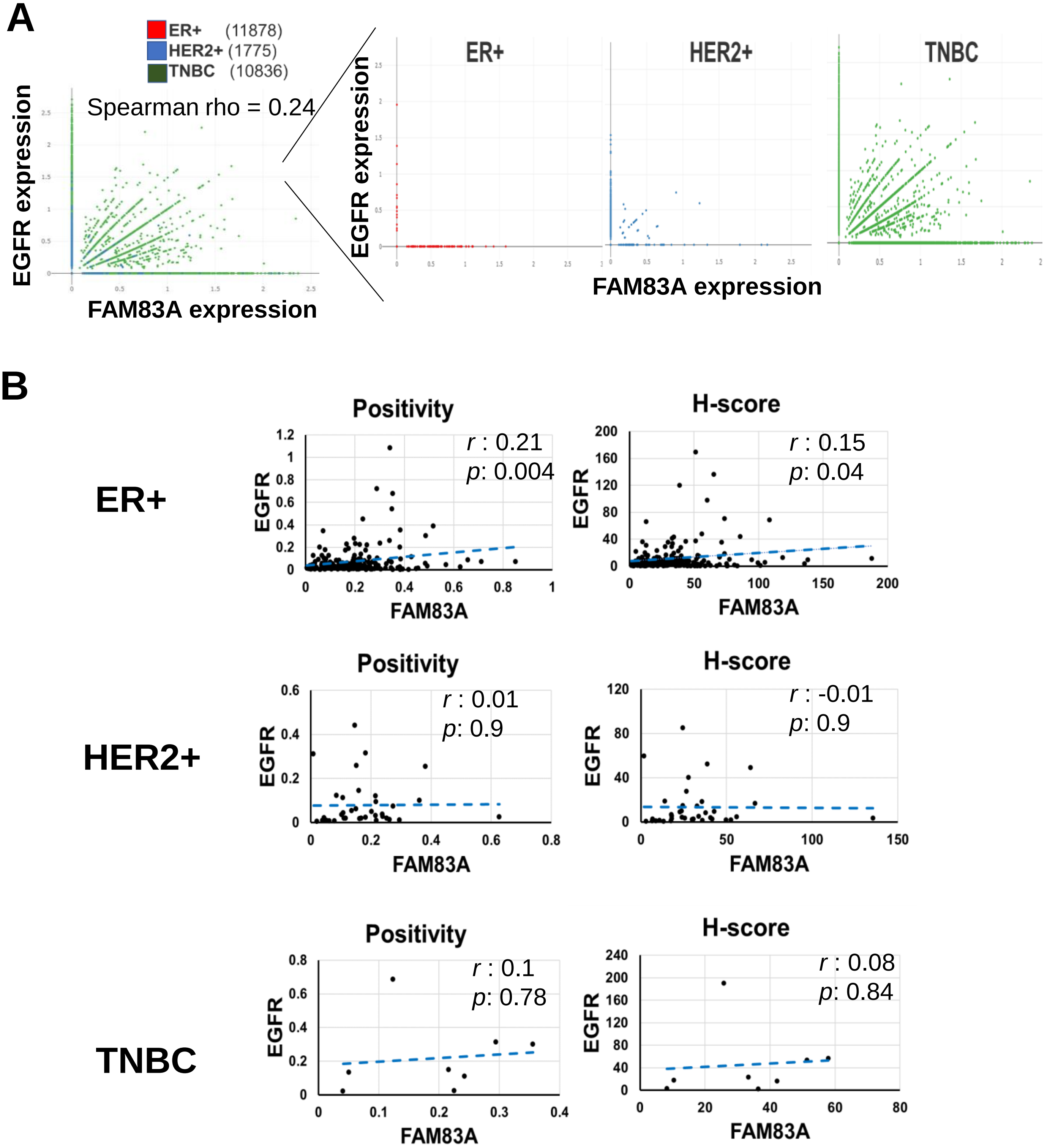


**Supplementary Figure 4**: **FAM83A-EGFR expression correlation in breast cancer subtypes.** (**A**) The Single Cell Portal (https://singlecell.broadinstitute.org/single_cell) was interrogated to evaluate FAM83A and EGFR expression and their direct correlation in epithelial cells from ER+, HER2+, and triple negative breast cancer tissues.(**B**) Correlation analysis of FAM83A and EGFR immunostaining level by breast cancer subtype. Pearson correlation coefficient (r ) and *p* values (*p*) are shown.
